# Molecular Diagnostics in Heart Failure: From Biomarkers to Personalized Medicine

**DOI:** 10.3390/diagnostics15141807

**Published:** 2025-07-17

**Authors:** Ovidiu Țica, Otilia Țica

**Affiliations:** 1 Department of Morphological Disciplines, Faculty of Medicine and Pharmacy,410073 Oradea, Romania; 2 Pathology Department, Emergency County Clinical Hospital of Bihor, 410165 Oradea, Romania; 3 Cardiology Clinic, Emergency County Clinical Hospital of Bihor, 410165 Oradea, Romania

**Keywords:** heart failure, molecular diagnostics, biomarkers, omics, precision medicine, liquid biopsy, genomics, proteomics, artificial intelligence, systems biology

## Abstract

Heart failure (HF) is a global health burden characterized by high morbidity and mortality, necessitating advancements in diagnostic and therapeutic approaches. Molecular diagnostics, encompassing genomics, transcriptomics, proteomics, metabolomics, and epigenetics, offer unprecedented insights into HF pathogenesis, aiding early diagnosis, risk stratification, and personalized management. This state-of-the-art review critically examines recent developments in molecular diagnostics in HF, evaluates their translational potential, and highlights key challenges in clinical implementation. Emerging tools such as liquid biopsy, multi-omics integration, and artificial intelligence (AI)-driven platforms are explored. We propose strategies to enhance clinical translation, equity in access, and utility in guiding treatment, thereby advancing precision cardiovascular medicine

## 1. Introduction

Heart failure (HF) affects over 64 million individuals globally and represents a leading cause of hospitalization, morbidity, and healthcare expenditure [1]. Heart failure is a complex clinical syndrome resulting from structural and/or functional cardiac abnormalities that impair the heart’s ability to pump blood efficiently. Despite substantial therapeutic advancements [2], particularly for patients with reduced ejection fraction (HFrEF), the prognosis remains dismal, with high rates of hospitalization, rehospitalization, and mortality. The situation is even more challenging for patients with preserved ejection fraction (HFpEF) [3], where effective therapies remain elusive.

Currently available clinical diagnostic tools, including imaging modalities (e.g., echocardiography, cardiac MRI) and circulating biomarkers like B-type natriuretic peptide (BNP) and N-terminal proBNP (NT-proBNP), are limited in their specificity and sensitivity. These conventional biomarkers mainly reflect hemodynamic stress and fluid overload but fail to capture the heterogeneity of HF pathogenesis, especially in early or ambiguous stages [4,5]. This diagnostic imprecision hampers personalized management and delays timely intervention.

This review critically examines the current landscape of molecular diagnostics in heart failure, evaluating both traditional and emerging biomarkers and omics technologies. This review explores their translational potential, highlights challenges in clinical adoption, and provides actionable recommendations for future research aimed at realizing the promise of precision cardiovascular medicine.

Molecular diagnostics offer an unprecedented opportunity to delve deeper into HF pathophysiology by uncovering the genetic, transcriptomic, proteomic, metabolomic, and epigenetic alterations that underlie disease onset and progression.

While molecular diagnostics indeed hold significant promise to transform heart failure (HF) care through early detection, personalized treatment, and enhanced risk stratification, the clinical translation of these tools remains in a formative stage. The identification of disease-specific molecular signatures and the integration of multi-omics data with AI-driven analytics [6,7,8,9] represent powerful conceptual advances, yet their real-world application is often hindered by challenges including lack of standardization, insufficient validation in diverse HF populations, and limited integration into prospective clinical trials.

## 2. Traditional and Emerging Biomarkers in Heart Failure

### 2.1. Cardiac Troponins and Natriuretic Peptides

Cardiac troponins (cTnI and cTnT) are highly specific and sensitive markers of myocardial injury, widely employed in both acute coronary syndrome and heart failure contexts [10,11] of myocardial injury and are increasingly used to risk-stratify HF patients [12,13,14]. In HF patients, especially those with HFrEF, elevated troponin levels are associated with worse outcomes, including higher mortality and hospitalization rates. Natriuretic peptides (BNP, NT-proBNP) remain pivotal in diagnosing HF and predicting adverse outcomes [15,16,17]. The advent of high-sensitivity assays has enabled the detection of even minor degrees of myocardial injury, improving prognostic stratification and enabling timely therapeutic interventions [18,19].

Natriuretic peptides, including BNP and NT-proBNP, are secreted in response to ventricular wall stress and volume overload. They remain foundational to HF diagnosis and management guidelines, endorsed by ESC and ACC/AHA/HFSA [20,21]. BNP levels correlate with HF severity and can be used to assess treatment response and predict readmission risk. Moreover, natriuretic peptides play a critical role in distinguishing HF from other causes of dyspnea in acute settings. Recent evidence supports their role not only in diagnosis but also in guiding therapy, as demonstrated in trials like GUIDE-IT [22], although outcomes remain variable based on patient heterogeneity.

Importantly, troponins and natriuretic peptides provide complementary insights—myocardial injury and hemodynamic stress, respectively—and are increasingly utilized in tandem to enhance diagnostic precision. Limitations include confounding effects from renal dysfunction, obesity, and atrial fibrillation, which necessitate the development of additional biomarkers and diagnostic algorithms.

Women and men with heart failure not only exhibit divergent clinical phenotypes—women more often presenting with HFpEF and men with HFrEF—but also require sex-specific biomarker reference ranges, as fertile women have higher baseline natriuretic peptide levels and lower 99th-percentile thresholds for high-sensitivity cardiac troponins than men, mandating the use of age- and sex-adjusted cutoff values to optimize diagnostic and prognostic accuracy [23,24].

### 2.2. Novel Circulating Biomarkers

Recent candidates such as Galectin-3 [25], soluble ST2 (sST2) [26], growth differentiation factor-15 (GDF-15), and copeptin reflect pathways including fibrosis, inflammation, and neurohormonal activation [27,28]. Multi-marker strategies integrating these biomarkers have improved prognostic accuracy [29,30]. However, analytical variability and lack of standardized thresholds limit clinical adoption.

**Figure 1:**
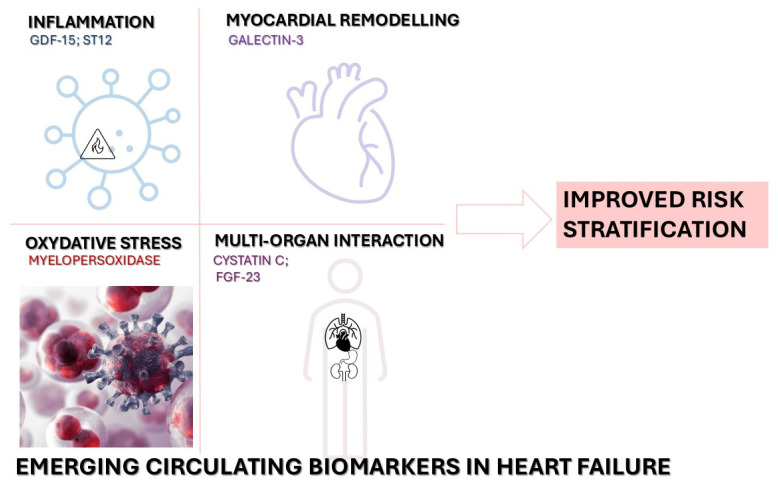
Emerging circulating biomarkers in heart failure. Examples of emerging circulating biomarkers in heart failure. These include markers of inflammation (GDF-15, ST2), myocardial remodeling (Galectin-3), oxidative stress (myeloperoxidase), and multi-organ interaction (cystatin C, FGF-23), which may improve risk stratification and reflect specific pathophysiological pathways. FGF-23, fibroblast growth factor-23; GDF-15, growth differentiation factor-15; ST2, soluble suppression of tumorigenicity-2.

Beyond traditional markers, novel circulating biomarkers have emerged to address the complex and multifactorial pathophysiology of HF, as the examples outlined in Figure 1. Galectin-3, a biomarker of fibrosis and inflammation, is secreted by activated macrophages and fibroblasts. Elevated levels of Galectin-3 are linked with adverse cardiac remodeling and have demonstrated prognostic utility in both HFpEF and HFrEF populations [15]. Similarly, sST2 is a decoy receptor for IL-33 and a marker of myocardial strain and inflammation [31]. High sST2 levels are associated with worse clinical outcomes and have been incorporated into risk stratification models for both acute and chronic HF [32].

GDF-15 is a cytokine induced by oxidative stress and mitochondrial dysfunction and is upregulated in various cardiovascular and non-cardiovascular pathologies. Elevated GDF-15 levels correlate with all-cause mortality in HF, particularly in elderly patients and those with comorbidities [33]. Copeptin, a stable peptide derived from the precursor of vasopressin, reflects neurohormonal activation and has shown prognostic value in acute decompensated HF and post-MI HF [34]. Additionally, heart-type fatty acid binding protein (H-FABP), a marker of myocardial ischemia, has demonstrated early diagnostic value in acute HF [35].

Emerging metabolic and inflammatory biomarkers such as fibroblast growth factor-23 (FGF-23), pentraxin-3 (PTX-3), and neprilysin have also shown recent promise [36]. FGF-23, primarily involved in phosphate regulation, is linked to left ventricular hypertrophy and adverse outcomes in HF patients [37]. PTX-3, an acute-phase protein, has demonstrated potential for predicting cardiovascular events, especially in inflammatory-driven HF pathogenesis [38]. Neprilysin, targeted pharmacologically by angiotensin receptor–neprilysin inhibitors (ARNIs), has gained attention not only as a therapeutic target but also as a biomarker of treatment response [39].

The incorporation of these biomarkers into multiplex panels or machine learning-based risk prediction models offers promise for precision diagnostics. However, their broader clinical adoption requires further validation through large-scale, multi-center prospective trials, assay standardization, and cost-effectiveness analyses. Combining these novel biomarkers with clinical, imaging, and genetic data may enable more nuanced phenotyping of HF and facilitate personalized therapeutic strategies.

We have outlined in Table 1 the advantages and limitations of each biomarker, emphasizing their roles in diagnosis and in monitoring the response to heart failure treatment. Table 1 summarizes the key advantages and limitations of individual circulating biomarkers and multi-marker strategies for diagnosing and monitoring heart failure, highlighting their pathophysiological roles, prognostic value, and practical constraints such as specificity, assay variability, and monitoring utility.

## 3. Genomics and Transcriptomics in HF Diagnostics

### 3.1. Genetic Risk and Polygenic Scores

Monogenic variants contribute to HF in familial dilated cardiomyopathy (DCM), whereas genome-wide association studies (GWAS) have identified polygenic contributors to common forms of HF [51,52,53]. Polygenic risk scores (PRS) may stratify susceptibility and inform prevention.

Genetic predisposition plays a central role in certain HF subtypes. In familial DCM, pathogenic mutations in genes such as TTN, LMNA, MYH7, and SCN5A are well-documented contributors. Next-generation sequencing (NGS) has facilitated the discovery of novel variants associated with DCM, restrictive cardiomyopathy, and arrhythmogenic cardiomyopathy [54]. GWAS [55] have identified multiple single-nucleotide polymorphisms (SNPs) associated with HF risk. PRS, aggregating SNP effects, are gaining traction for individualized HF risk prediction.

### 3.2. Transcriptomic Profiling

RNA sequencing has identified differential gene expression signatures between HF subtypes and stages [56]. Upregulation of inflammatory and fibrotic pathways characterizes both HFrEF and HFpEF. Microarray and single-cell RNA-sequencing (ScRNA-seq) analyses continue to elucidate cell-specific responses in HF pathology.

Transcriptomics provides insights into gene expression dynamics associated with HF development and progression. Differential gene expression helps distinguish HF phenotypes and reveals dysregulated pathways, such as inflammation, oxidative stress, and fibrosis. ScRNA-seq has revolutionized our understanding of the myocardial microenvironment, uncovering cell-specific expression profiles and intercellular communication networks that drive HF pathology. Non-coding RNAs, including miRNAs and lncRNAs, have emerged as key regulators and potential biomarkers.

Bulk RNA sequencing has been instrumental in identifying global transcriptional changes in heart failure, yet it averages signals across diverse cell types, potentially masking cell-specific responses. In contrast, single-cell RNA sequencing (scRNA-seq) offers higher resolution by capturing transcriptomic heterogeneity at the cellular level, enabling the discovery of rare cell populations and intercellular signaling networks central to HF pathogenesis [57]. Integrating both approaches provides complementary insights into disease mechanisms and biomarker development.

## 4. Epigenetics and Non-Coding RNAs

Epigenetic modifications, including DNA methylation, histone modifications, and non-coding RNAs, regulate gene expression without altering DNA sequence [58]. These modifications respond dynamically to environmental and pathological cues, contributing to HF onset and progression. Aberrant DNA methylation patterns in cardiac tissues have been associated with adverse remodeling and inflammation. Histone acetylation influences chromatin structure and transcriptional activity in failing myocardium [59]. Circulating epigenetic markers offer non-invasive tools for diagnosis and prognostication. Altered methylation of genes like NPPA and HAND2 and dysregulated microRNAs (miRNAs) (e.g., miR-21, miR-423-5p) are implicated in HF remodeling [60,61]. Circulating miRNAs are emerging as minimally invasive diagnostic biomarkers.

## 5. Proteomics and Metabolomics

### 5.1. Proteomic Insights

Proteomic platforms such as mass spectrometry and aptamer-based assays have revealed dynamic changes in proteins related to extracellular matrix remodeling, oxidative stress, and mitochondrial function in HF [62,63,64]. Protein panels may outperform single biomarkers in predicting outcomes.

Proteomic and metabolomic approaches offer complementary insights into HF pathophysiology. Proteomics enables the profiling of protein networks and post-translational modifications, revealing candidates involved in remodeling, hypertrophy, and inflammation. Mass spectrometry and aptamer-based platforms have identified protein panels predictive of adverse outcomes [65]. Metabolomics characterizes low-molecular-weight metabolites reflecting cellular metabolism, energy imbalance, and oxidative stress [66]. Profiling of amino acids, lipids, and ketone bodies distinguishes HFpEF from HFrEF and predicts decompensation [8]. Integration of proteomic and metabolomic data offers powerful stratification tools for personalized therapy.

### 5.2. Metabolomic Profiling

Metabolomics identifies small molecule perturbations in HF, including altered amino acid, lipid, and energy metabolism [67,68]. Acylcarnitine [69] and ketone bodies [70] have been linked to disease severity and response to therapy.

## 6. Biopsy and Non-Invasive Molecular Tools

Liquid biopsy technologies detect circulating cell-free DNA (cfDNA), RNA, exosomes, and extracellular vesicles, reflecting cardiac pathology in real time [71,72]. These tools offer advantages in longitudinal monitoring and patient stratification, especially in HF patients undergoing advanced therapies or transplantation.

**Figure 2:**
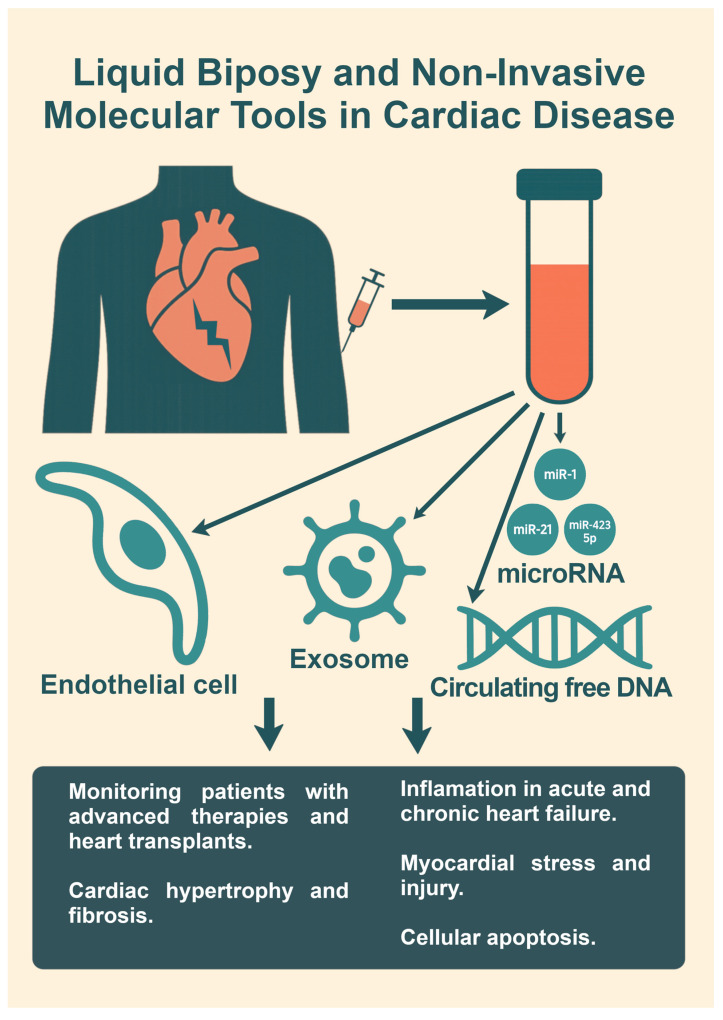
Liquid biopsy and non-invasive molecular tools in cardiovascular disease. The image illustrates how liquid biopsy technologies—such as cfDNA, RNA, microRNAs, and exosomes—enable non-invasive, real-time monitoring of cardiac pathology in heart failure, aiding in diagnosis, patient stratification, and disease tracking.

Liquid biopsy, the sampling and analysis of non-solid biological tissues such as blood, provides a non-invasive and dynamic window into the molecular landscape of heart failure, as revealed in Figure 2. Initially developed in oncology [73], this technique has gained traction in cardiology due to its ability to detect and monitor circulating biomarkers like cell-free DNA (cfDNA), RNA species (including miRNAs), exosomes, and circulating tumor or endothelial cells [74]. These molecular signatures offer real-time insight into cardiac injury, remodeling, inflammation, and even treatment response, facilitating longitudinal patient monitoring without the need for repeated tissue biopsies.

One of the most promising elements of liquid biopsy in HF is the detection of circulating miRNAs [75,76], which regulate gene expression post-transcriptionally and are dysregulated in various HF phenotypes. For instance, miR-1, miR-21 [77], and miR-423 [78] have shown consistent associations with cardiac hypertrophy, fibrosis, and myocardial stress. Additionally, exosomes—nanovesicles carrying proteins, lipids, and nucleic acids—are now recognized as vehicles of intercellular communication and reflect the pathological state of their cell of origin. In HF, exosome-derived miRNAs and proteins serve as sensitive markers of cardiac stress and may even modulate disease progression.

Circulating cfDNA, which may contain cardiac-specific methylation patterns or gene mutations, is emerging as a novel diagnostic and prognostic marker. For example, increased levels of cfDNA [79] have been linked to myocardial injury, apoptosis, and inflammation in acute and chronic HF settings. Further, transcriptomic profiling of extracellular RNA (exRNA) [79] from blood plasma has opened avenues for discovering non-invasive gene expression signatures linked to adverse cardiac remodeling and clinical outcomes.

Despite its promise, several limitations impede the clinical translation of liquid biopsy in HF. These include the lack of standardized protocols for sample collection, processing, and analysis; the need for robust validation studies; and the requirement for integrating liquid biopsy data with existing clinical and imaging information. Nevertheless, the rapid evolution of omics technologies and machine learning models is likely to overcome these barriers, paving the way for a new era of non-invasive molecular diagnostics in HF.

Pre-analytical variables such as sample type, collection timing, processing delays, and storage conditions significantly influence molecular assay performance and biomarker reliability. Inconsistent handling can lead to RNA degradation, protein denaturation, or cfDNA fragmentation, ultimately affecting reproducibility and clinical interpretation. Moreover, cross-platform discrepancies—particularly in transcriptomic and proteomic analyses—highlight the need for rigorous standardization and validation across laboratories and technologies [80].

In heart transplant recipients, serial measurement of circulating biomarkers—such as donor-derived cfDNA, exosome-encapsulated miRNAs, and inflammatory proteins—can detect graft injury and subclinical rejection earlier than biopsy alone, and when combined in multi-marker panels, they guide tailored immunosuppression; integrating these molecular signatures with cardiac CT–derived pericoronary fat attenuation index (FAI) further enhances specificity by quantifying peri-adipose inflammation, offering a minimally invasive, multimodal strategy to personalize rejection surveillance and improve outcomes [81].

## 7. Artificial Intelligence and Systems Biology Approaches

Machine learning (ML) algorithms are increasingly used to integrate omics data with clinical and imaging inputs [82]. Predictive models have identified HF sub-phenotypes [9] with distinct outcomes. Systems biology approaches map complex molecular networks, revealing novel therapeutic targets [83].

AI and ML are revolutionizing [84] the interpretation of high-dimensional molecular data, representing a transformative force in cardiovascular diagnostics. These computational methodologies enable the integration and analysis of vast datasets generated from genomics, proteomics, transcriptomics, epigenetics, metabolomics, and imaging platforms to derive predictive, prognostic, and diagnostic insights with enhanced accuracy. AI can discern subtle patterns often undetectable to human analysis, offering potential for early HF diagnosis, risk stratification, and therapeutic response monitoring.

ML algorithms such as random forests, support vector machines, and neural networks [85] have been used to identify novel biomarker signatures, predict HF hospitalization and mortality, and classify HF phenotypes [9], including the challenging HFpEF subtypes. Deep learning techniques, particularly convolutional neural networks, have enabled automated interpretation of echocardiographic [85] and cardiac MRI data, improving diagnostic precision and standardization. Integration with ECG data has allowed ML models to detect molecular and structural abnormalities, bridging phenotypic and genotypic diagnostics.

**Figure 3:**
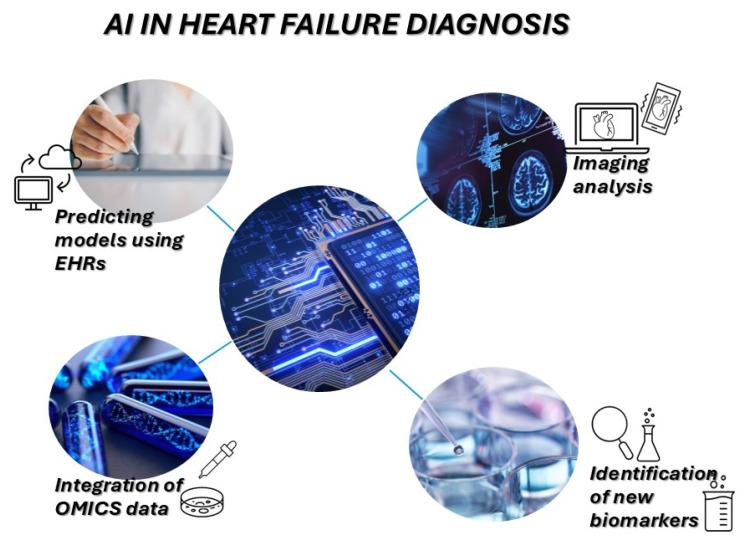
AI applications in heart failure diagnostics. Schematic illustration of AI applications in heart failure diagnostics, including predictive modeling using EHRs, imaging analysis, integration of omics data, and identification of new biomarkers. AI, artificial intelligence; EHRs, electronic health records.

Moreover, AI-powered clinical decision support systems (CDSS) are being embedded into electronic health records (EHRs) [86] to provide real-time, data-driven guidance for personalized therapy selection, as seen in Figure 3. These systems can incorporate omics data to refine drug selection, anticipate adverse effects, and optimize care pathways. Natural language processing (NLP) further enhances AI’s ability to synthesize unstructured clinical notes, contributing to comprehensive risk profiles.

However, several challenges hinder full-scale implementation. Data heterogeneity, lack of standardized input formats, algorithmic bias due to unbalanced training datasets, and limited explainability remain critical obstacles [87]. Regulatory approval pathways for AI-based tools are evolving, but still unclear [88,89], especially for black-box models [90]. Furthermore, clinical skepticism and a lack of training in AI interpretation can impede adoption. Future priorities must include the development of interpretable, transparent AI models, external validation using diverse and representative cohorts, and the establishment of ethical frameworks governing data use. Cross-disciplinary collaboration between clinicians, data scientists, and regulatory bodies is essential to unlock AI’s full potential in HF molecular diagnostics. A key concern with AI-driven biomarker models is the risk of overfitting, where algorithms perform exceptionally on development datasets but significantly underperform when applied to new, independent cohorts due to cohort-specific biases or small sample sizes. This lack of generalizability is particularly problematic: a recent cross-sectional analysis of 903 FDA-approved AI devices found that only about 56% reported any clinical performance study at approval, and just 8% were validated prospectively—many lacked demographic subgroup analyses altogether [88]. Additionally, the prevalence of black-box models, which offer little transparency into their decision making, raises serious concerns about clinical trust and safety; their opacity challenges regulatory scrutiny and clinician adoption. Finally, although regulatory bodies like the FDA and EU have issued guidance on AI/ML-enabled devices, comprehensive regulatory oversight—including post-market surveillance, bias mitigation, and adaptive learning management—remains fragmented and inconsistent, jeopardizing safe clinical deployment [91].

Ensemble tree-based models often excel on structured clinical and laboratory data: for example, a random forest classifier achieved a specificity of 0.93 and an AUC of 0.97 for heart failure classification [92]. In contrast, convolutional neural networks outperform traditional methods on imaging tasks—one CNN distinguished five standard echocardiographic views with 98.1% accuracy (AUC 0.97)—but demand large, well-annotated datasets and frequently underperform when applied across different scanners or centers [93]. Natural-language-processing pipelines, such as the C3PO model for adjudicating HF hospitalizations, maintain high sensitivity (~94%) but see Cohen’s κ drop from 0.85 (inter-rater) to 0.69 on external validation, underscoring vocabulary drift and portability issues [94]. Finally, although model-agnostic explainability tools (e.g., SHAP, LIME) are increasingly applied, scoping reviews highlight inconsistent transparency, safety reporting, and regulatory guidance for AI/ML-enabled medical devices [91,95]. To translate AI from promise to practice, the field must adopt standardized multi-center benchmarking, adhere to reporting frameworks (e.g., TRIPOD-AI and CONSORT-AI), and integrate rigorous explainability and validation metrics.

While artificial intelligence (AI) and multi-omics integration are rapidly advancing cardiovascular research, current studies often fall short in methodological rigor, external validation, and clinical reproducibility. A recent paper [82] proposes a promising framework for integrating multi-omics data using machine learning to uncover disease pathways and biomarkers; however, these models are frequently developed on small or homogeneous datasets, limiting their applicability to broader, real-world populations. Similarly, recent studies [83,85] demonstrate accurate prediction of clinical endpoints, such as left ventricular ejection fraction, using neural networks, yet these models often lack rigorous external validation and tend to underperform in independent cohorts with different demographic and clinical profiles. Although there are highlights that promise AI–human synergy [84], recent analyses reveal substantial barriers. For example, it was found that many AI-enabled tools in cardiovascular trials are inconsistently evaluated, with limited transparency in algorithm development and outcome reporting [87]. Moreover, a systematically assessed FDA-approved AI tool concluded that generalizability across populations and clinical settings remains a major concern [88]. Regulatory evaluations [89] further underscore the fragmented landscape of AI oversight in healthcare across the EU, emphasizing the urgent need for standardized validation protocols, real-world testing, and longitudinal assessment. Collectively, while the cited literature reflects substantial innovation, it also illustrates key limitations in reproducibility, transparency, and scalability, highlighting the need for robust, multi-center validation before clinical integration.

## 8. Clinical Translation: Challenges and Opportunities

Despite promising discoveries, several barriers hinder the translation of molecular diagnostics into routine care. These include high assay costs, limited standardization, data interpretation complexity, and regulatory uncertainties [96,97]. Collaborative consortia and real-world validation studies are needed to establish clinical utility.

Bridging the gap between molecular discovery and clinical implementation requires comprehensive validation studies, cost-effectiveness analyses, and the establishment of translational frameworks. Clinical trials incorporating biomarker-based stratification, such as GUIDE-IT (which assessed NT-proBNP-guided therapy) [98] and BIOSTAT-CHF (focusing on personalized treatment algorithms) [99], have demonstrated the clinical utility of integrating molecular diagnostics into heart failure (HF) management. These studies underscore the potential of biomarkers in optimizing therapy, monitoring disease progression, and improving patient outcomes. Although GUIDE-IT aimed to demonstrate the benefit of NT-proBNP-guided therapy, it was stopped early due to futility, raising questions about the incremental value of biomarker-guided care over standard clinical management [98]. Similarly, while BIOSTAT-CHF offered insights into personalized treatment algorithms, its observational design and European-centric cohort may limit generalizability to more diverse populations [99].

Currently, molecular diagnostics are increasingly being used to guide pharmacological therapy by identifying patients who may benefit from specific agents, such as ARNi, sodium–glucose cotransporter-2 (SGLT2) inhibitors, and mineralocorticoid receptor antagonists (MRAs). For instance, genomic profiling can help recognize responders and non-responders to certain drugs, minimizing adverse effects and improving therapeutic efficacy [100]. Molecular testing has also informed decisions around advanced interventions, including cardiac resynchronization therapy (CRT), left ventricular assist devices (LVADs), and even gene therapy in genetically defined cardiomyopathies.

Despite these advancements, widespread clinical integration of omics data is limited by several factors [82,101]. These include lack of interoperability across health IT systems, inadequate training of clinicians in molecular medicine, high cost and complexity of data analysis, and absence of streamlined clinical decision pathways. Furthermore, many molecular assays are not yet validated in large, diverse patient populations, raising concerns about generalizability and equity.

Despite extensive efforts, omics-derived biomarkers in heart failure have often faltered when moving from discovery into clinical practice. For example, a transcriptomic signature designed to distinguish dilated from ischemic cardiomyopathy failed to replicate in one of four external cohorts despite robust discovery-phase performance [102]. Similarly, metabolomic profiles linked to incident heart failure in the Framingham Heart Study offered only marginal incremental predictive value over traditional risk factors, with negligible gains in discrimination [103]. Furthermore, circulating microRNAs have yielded inconsistent prognostic associations—systematic reviews identify marked heterogeneity across studies and only a handful of miRNAs (e.g., miR-423-5p, miR-122) appear in more than one cohort, none meeting criteria for clinical deployment [104]. These negative findings underscore the necessity of rigorous experimental design, standardized pre-analytical workflows, and validation in large, diverse cohorts before omics biomarkers can be translated into personalized molecular diagnostics for heart failure.

Underrepresentation of diverse populations in genomic and multi-omics studies can compromise biomarker validity and limit the generalizability of AI models, leading to reduced diagnostic accuracy and potential inequities in care delivery [105]. Moreover, ethical concerns around data ownership, re-identification, and genetic privacy require structured frameworks, including dynamic consent models and robust anonymization protocols, to ensure transparency, trust, and equitable access in precision medicine.

To overcome these barriers, health systems must invest in infrastructure such as centralized diagnostic hubs and molecular tumor board-style multi-disciplinary teams for cardiovascular care. These teams would consist of cardiologists, molecular pathologists, genetic counsellors, bioinformaticians, and clinical pharmacologists working collaboratively to interpret omics results and formulate patient-specific management plans. Initiatives aimed at clinician education, development of intuitive bioinformatics tools, and incorporation of molecular data into EHRs with actionable alerts are also pivotal.

Ultimately, a successful translational ecosystem will depend on harmonized clinical guidelines, public-private partnerships, and robust regulatory pathways to ensure safe, ethical, and effective integration of molecular diagnostics into routine HF care.

## 9. Limitations

Molecular diagnostics are revolutionizing our understanding of HF heterogeneity. However, few tools have crossed the bench-to-bedside divide. Integration into electronic health records, clinician education, and cost-effectiveness analyses are essential next steps. Personalized HF management depends on comprehensive phenotyping enabled by molecular tools.

Despite exciting progress, several barriers hinder the widespread adoption of molecular diagnostics in HF [106]. Standardization of assay techniques remains a major challenge, as inter-laboratory variability and inconsistent assay sensitivity affect reproducibility and clinical reliability. Without uniform protocols for sample collection, processing, and analysis, results may vary across institutions, complicating multi-center collaborations and meta-analyses.

Another key limitation is the lack of consensus on biomarker thresholds and interpretation. For many emerging biomarkers, cutoff values for clinical action are not well defined or validated in diverse populations, hampering their integration into treatment algorithms. Additionally, many omics-based tools require sophisticated data processing pipelines and expert interpretation, making them inaccessible for routine use in many clinical settings.

The cost and complexity of high-throughput platforms such as NGS [107], mass spectrometry, and advanced imaging technologies continue to be a barrier, especially in low- and middle-income countries. These technologies often require specialized personnel, infrastructure, and bioinformatics support, which are not widely available outside academic or tertiary centers.

Ethical and legal considerations add further complexity. Genomic data pose unique challenges [108] around patient consent, privacy, and the handling of incidental findings. Issues such as genetic discrimination, data ownership, and intergenerational implications of testing remain unresolved in many healthcare systems [109]. Similarly, the use of AI in diagnostics raises questions about algorithm transparency, liability, and trustworthiness.

Furthermore, disparities in data availability across populations, particularly underrepresentation of ethnic minorities, introduce significant bias and reduce the generalizability of findings [110]. Many biomarker studies are conducted on small, homogenous cohorts and lack sufficient longitudinal data to assess long-term predictive value. The need for larger, more inclusive datasets and multi-ethnic validation studies is urgent. Furthermore, the majority of molecular diagnostic tools are still in the research or early validation phase, with few having reached regulatory approval for clinical use.

Health economic evaluations are also critical and are urgently needed to justify payer reimbursement and determine cost-effectiveness in real-world healthcare settings [111]. Reimbursement for molecular diagnostic tests is often limited, and there is insufficient evidence of cost-effectiveness in real-world settings. To inform policy and payer decisions, studies must assess not just clinical efficacy but also budget impact, health outcomes, and system-wide efficiency [112]. In addition, data privacy and ethical concerns related to genomic testing—including the management of incidental findings and familial implications—require standardized consent processes and data governance frameworks. Clinician and patient education on the benefits, limitations, and ethical implications of molecular diagnostics is also lacking.

To address these limitations, future research should prioritize (1) harmonizing assay platforms and reference standards; (2) expanding biomarker discovery efforts to include diverse and underrepresented populations; (3) developing affordable, portable diagnostic devices suitable for decentralized or point-of-care settings; (4) conducting robust multi-center trials to validate biomarker utility and economic viability; (5) establishing governance structures for data privacy and ethical use. Building infrastructure for data sharing, clinician education, and cross-disciplinary collaboration will also be key to overcoming current challenges and ensuring equitable integration of molecular diagnostics into HF care.

## 10. Future Directions

The future of molecular diagnostics in heart failure lies in the integration of multi-omics data with advanced analytics to achieve truly personalized care [7]. As omics technologies become more accessible and cost-effective, comprehensive profiling of genomic, transcriptomic, proteomic, and metabolomic signatures will enable clinicians to identify patient-specific disease mechanisms and tailor therapeutic strategies accordingly. The implementation of systems biology approaches will facilitate a more holistic understanding [113] of HF as a network-based disease rather than a condition driven by single pathways or markers.

Furthermore, advances in machine learning and artificial intelligence will play a critical role in interpreting high-dimensional omics data, identifying novel biomarkers, and predicting clinical outcomes. Future research should focus on developing robust, externally validated prediction models that integrate molecular, imaging, and clinical data for improved phenotyping and treatment decision making [114]. Longitudinal cohort studies, real-world evidence, and multi-center trials are essential to validate the clinical utility of emerging biomarkers and technologies.

Several ongoing and completed clinical trials listed in ClinicalTrials.gov reflect this shift towards precision medicine in HF. For instance, the DEFINE-HF trial (NCT02693509) [68] and PARAGLIDE-HF (NCT03988634) [115] incorporate biomarker-driven endpoints, while the PROMINENT-HF study (NCT05187003) evaluates multi-omics-based algorithms for therapy guidance. Other notable studies, such as BACE1AS-HF(NCT06213493) that are investigating the use of cfDNA and RNA as liquid biopsy tools in risk stratification. In Table 2, we have included ongoing and recent clinical trials investigating molecular diagnostics in heart failure. These initiatives exemplify how biomarker integration into clinical practice is being actively pursued and evaluated across international cohorts. These trials represent the future direction of heart failure diagnostics, integrating molecular tools with AI-based analytics to advance personalized medicine.

Additionally, the democratization of molecular diagnostics through point-of-care testing and portable sequencing platforms could bridge healthcare disparities and expand access in resource-limited settings. Ethical considerations, data privacy, and equitable implementation must also be addressed to ensure that innovations benefit all patient populations. In summary, future efforts should emphasize multi-disciplinary collaboration, standardization of methodologies, and translational research to bridge the gap between bench discoveries and bedside [119] application in HF management [120].

## 11. Conclusions

The integration of molecular diagnostics into HF care holds transformative potential. By combining biomarkers, omics, and AI analytics, clinicians can move towards truly personalized medicine. Continued investment in translational research, infrastructure, and equitable implementation is required.

## Figures and Tables

**Table 1:** Advantages and limitations of circulating biomarkers and multi-marker strategies in heart failure diagnosis and monitoring.

Biomarker/Strategy	Advantages	Limitations
Galectin-3 [25]	Reflects fibrosis and inflammation; secreted by activated macrophages/fibroblasts; Linked to adverse remodeling in HFpEF and HFrEF; Independent prognostic marker for mortality and rehospitalization	Influenced by renal dysfunction and other fibrotic diseases, reducing specificity; Limited dynamic change with therapy, so less useful for monitoring response; No universally accepted assay thresholds.
Soluble ST2 (sST2) [40]	Marker of myocardial strain and inflammation (IL-33 decoy receptor); Adds prognostic value beyond NP and troponin in acute and chronic HF; Less affected by age, obesity, or renal function than natriuretic peptides.	Assay cost and availability barriers; Cutoffs vary between platforms; no standard thresholds; Elevated in systemic inflammatory states, reducing cardiac specificity.
GDF-15 [41,42]	Reflects oxidative stress and mitochondrial dysfunction; Correlates with all-cause mortality, especially in elderly/comorbid HF patients; Provides incremental prognostic information when combined with NP and troponin.	Highly non-specific (elevated in malignancy, renal disease, inflammation); Limited data on serial changes with therapy; Not yet part of routine HF management algorithms.
Copeptin [43]	Surrogate for vasopressin release; reflects neurohormonal activation; Prognostic in acute decompensated HF and post-MI HF; Stable peptide, easier to measure than vasopressin.	Rises with any systemic stress (sepsis, stroke), limiting cardiac specificity; Assay variability; no consensus on clinical cutoffs; Role in chronic HF monitoring remains undefined.
H-FABP [44]	Early marker of myocardial ischemia; appears rapidly after injury; May aid rapid diagnosis of acute HF in ED settings.	Very short half-life; timing critical; Cross-reactivity with skeletal muscle FABP can confound results; Limited prognostic value in stable, chronic HF.
FGF-23 [45,46]	Involved in phosphate regulation; elevated in HF and linked to LV hypertrophy; Associated with adverse outcomes; may identify cardio-renal axis dysfunction.	Strongly influenced by chronic kidney disease; Assay standardization lacking; variable thresholds; Limited evidence for therapy-monitoring utility.
Pentraxin-3 (PTX-3) [45,47]	Acute-phase protein reflecting vascular and myocardial inflammation; Predicts CV events, particularly in inflammation-driven HF.	Rises in any acute inflammatory state, limiting specificity; Sparse data on serial changes with HF treatment; No widely adopted assay or interpretive ranges.
Neprilysin [48]	Enzyme targeted by ARNIs; circulating levels may reflect neurohormonal balance and response to therapy; Potential dual role as biomarker and therapeutic target.	Assay complexity and lack of standardization; Directly modulated by ARNI therapy, complicating interpretation; Clinical thresholds not established; utility beyond research unproven.
Multi-marker strategies [49,50]	Integration of NP, troponin, sST2, Galectin-3, GDF-15, etc., improves risk stratification and prognostic accuracy; Captures multiple pathophysiological axes (hemodynamic stress, fibrosis, inflammation, neurohormonal activation).	Increased cost and logistical complexity; Analytical variability and lack of harmonized panels limit clinical adoption; No consensus on which combinations/algorithms to use in routine care.

ARNIs: angiotensin receptor–neprilysin inhibitors; CV: cardiovascular; FGF-23: fibroblast growth factor-23; GDF-15: growth differentiation factor-15; H-FABP: heart-type fatty acid-binding protein; HFpEF: heart failure with preserved ejection fraction; HFrEF: heart failure with reduced ejection fraction; LV: left ventricular; NP: natriuretic peptides; PTX-3: pentraxin-3; sST2: soluble suppression of tumorigenicity-2 (soluble ST2).

**Table 2:** Ongoing and recent clinical trials investigating molecular diagnostics and precision medicine in heart failure.

Acronym/Short Name	Title	Phase	Biomarker/Tool Investigated	Country	Intervention/ Treatment	Population	Primary Outcome	Reference
DCM Precision Medicine Study *	Genetic Testing and Cardiovascular Magnetic Resonance Imaging in Dilated Cardiomyopathy	N/A	Genetic testing, CMR imaging	USA	Genetic testing and CMR imaging	Patients with idiopathic dilated cardiomyopathy	Identification of myocardial scar and etiology	NCT03037632 [116]
HERMES *	Effects of Ziltivekimab Versus Placebo on Morbidity and Mortality in Patients With Heart Failure With Mildly Reduced or Preserved Ejection Fraction and Systemic Inflammation	Phase 3	High-sensitivity C-reactive protein (hsCRP), interleukin-6 (IL-6)	International	Ziltivekimab (IL-6 inhibitor)	Patients with HFpEF/HFmrEF and systemic inflammation	Cardiovascular death or heart failure hospitalization	NCT05636176
ZEUS *	Effects of Ziltivekimab Versus Placebo on Cardiovascular Outcomes in Participants With Established Atherosclerotic Cardiovascular Disease, Chronic Kidney Disease and Systemic Inflammation	Phase 3	hsCRP, IL-6	International	Ziltivekimab	Patients with atherosclerotic cardiovascular disease, chronic kidney disease, and systemic inflammation	Major adverse cardiovascular events (MACE)	NCT05021835
ATTRibute-CM *	Efficacy and Safety of Acoramidis in Transthyretin Amyloid Cardiomyopathy	Phase 3	Transthyretin (TTR) stabilization	International	Acoramidis (TTR stabilizer)	Patients with transthyretin amyloid cardiomyopathy (ATTR-CM)	Composite of all-cause mortality and cardiovascular-related hospitalization	NCT03860935
CUPID 2 ^†^	Calcium Upregulation by Percutaneous Administration of Gene Therapy in Cardiac Disease	Phase 2b	SERCA2a gene therapy	USA	Mydicar (AAV1/SERCA2a gene therapy)	Patients with advanced heart failure	Time to recurrent heart failure-related events	NCT01643330 [117]
RESCUE-2 ^†^	Efficacy and Safety of Interleukin-6 Inhibition With Ziltivekimab in Patients at High Risk of Atherosclerotic Events in Japan	Phase 2	hsCRP, IL-6	Japan	Ziltivekimab	Patients with chronic kidney disease and systemic inflammation	Reduction in hsCRP levels	NCT04626505
CDR132L ^†^	Safety and Tolerability of CDR132L in Patients With Heart Failure	Phase 1b	miR-132 levels	Germany	CDR132L (antisense oligonucleotide targeting miR-132)	Patients with heart failure	Safety and tolerability	NCT04045405 [118]

* Recruiting; ^†^ completed; ATTR-CM, transthyretin amyloid cardiomyopathy; CDR132L, antisense oligonucleotide targeting miR-132; CMR, cardiac magnetic resonance; hsCRP, high-sensitivity C-reactive protein; HFpEF, heart failure with preserved ejection fraction; HFrEF, heart failure with mildly reduced; IL-6, interleukin-6; MACE, major adverse cardiovascular events; TTR, transthyretin; USA, United States of America.

## Data Availability

Not applicable.

## Abbreviations

ACC	American College of Cardiology
AHA	American Heart Association
AI	artificial intelligence
ARNIs	angiotensin receptor–neprilysin inhibitors
BNP	B-type natriuretic peptide
CDSS	clinical decision support systems
cfDNA	cell-free DNA
CRT	cardiac resynchronization therapy
cTnI	cardiac troponins I
cTnT	cardiac troponins T
DCM	dilated cardiomyopathy
DNA	deoxyribonucleic acid
EHRs	electronic health records
ESC	European Society of Cardiology
exRNA	extracellular RNA
FGF-23	fibroblast growth factor-23
GDF-15	growth differentiation factor-15
GWAS	genome-wide association studies
HF	heart failure
H-FABP	heart-type fatty acid binding protein
HFpEF	heart failure with preserved ejection fraction
HFrEF	heart failure with reduced ejection fraction
HFSA	Heart Failure Society of America
IT	information technology
lncRNAs	long non-coding RNA
LVADs	left ventricular assist devices
MI	myocardial infarction
miRNAs	micro-RNA
ML	machine learning
MRAs	mineralocorticoid receptor antagonists
MRI	magnetic resonance imaging
NGS	next-generation sequencing
NLP	natural language processing
NT-proBNP	N-terminal proB-type natriuretic peptide
PRS	polygenic risk scores
PTX-3	pentraxin-3
RNA	ribonucleic acid
scRNA-seq	single-cell RNA sequencing
SGLT2	sodium–glucose cotransporter-2
SNPs	single-nucleotide polymorphisms
sST2	soluble suppression of tumorigenicity-2
